# Analysis of necroptosis-related prognostic genes and immune infiltration in idiopathic pulmonary fibrosis

**DOI:** 10.3389/fimmu.2023.1119139

**Published:** 2023-03-27

**Authors:** Hongzuo Chen, Zhenkun Xia, Bei Qing, Wei Wang, Linguo Gu, Ying Chen, Juan Wang, Yunchang Yuan

**Affiliations:** Department of Thoracic Surgery, The Second Xiangya Hospital of Central South University, Changsha, China

**Keywords:** necroptosis, immune filtration, idiopathic pulmonary fibrosis, bioinformatics analysis, prognostic prediction model

## Abstract

**Background:**

IPF is an undetermined, progressive lung disease. Necroptosis is a type of programmed apoptosis, which involved in the pathogenesis of lung diseases like COPD and ARDS. However, necroptosis in IPF have not been adequately studied. This study aimed to investigate the necroptosis in IPF and the relationship between necroptosis and immune infiltration, to construct a prognostic prediction model of IPF based on necroptosis-related genes.

**Methods:**

GSE110147 was downloaded from the GEO database and utilized to analyze the expression of necroptosis-related differentially expressed genes (NRDEGs). Then NRDEGs were used to construct protein-protein interaction (PPI) networks in the STRING database, and Cytoscape software was used to identify and visualize hub genes. Necroptosis-related prognosticgenes were explored in GSE70866, and a prognostic prediction model was constructed. The ImmuCellAI algorithm was utilized to analyze the landscape of immune infiltration in GSE110147. The single-cell RNA sequencing dataset GSE122960 was used to explore the association between necroptosis and type II alveolar epithelial cells (AT II) in IPF. The GSE213001 and GSE93606 were used for external validation. The expression of prognostic genes was quantified using RT-qPCRin the IPF A549 cell model, and was further verified by western blotting in the bleomycin-induced pulmonary fibrosis mouse model.

**Results:**

It was observed that necroptosis-related signaling pathways were abundantly enriched in IPF. 29 NRDEGs were screened, of which 12 showed consistent expression trends in GSE213001. Spearman correlation analysis showed that the expression of NRDEGs was positively correlated with the infiltration of proinflammatory immune cells, and negatively correlated with the infiltration of anti-inflammatory immune cells. NRDEGs, including MLKL, were highly expressed in AT II of fibrotic lung tissue. A necroptosis-related prediction model was constructed based on 4 NRDEGsby the cox stepwise regression. In the validation dataset GSE93606, the prognostic prediction model showed good applicability. The verification results of RT-qPCR and western blotting showed the reliability of most of the conclusions.

**Conclusions:**

This study revealed that necroptosis existed in IPF and might occur in AT II. Necroptosis was associated with immune infiltration, suggesting that necroptosis of AT II might involve in IPF by activating immune infiltration and immune response.

## Introduction

Idiopathic pulmonary fibrosis (IPF) is an undetermined, progressive lung disease. Patients with IPF have an average lifespan of about 3-5 years after diagnosis without treatment (1). In recent years, it has been noted that the incidence of IPF increasing over time (2–4). Given the poor prognosis of IPF, several IPF prognostic staging systems based on clinical indicators have been established (5–8). However, little is known about whether biomarkers of molecular events during IPF progression can predict IPF prognosis. Molecular biomarkers can be identified from bronchoalveolar lavage (BAL) cells of IPF patients, and BAL cell collection is non-invasive compared with lung biopsy (9). Considering the rapidity of pulmonary fibrosis progression, there is an urgent need for a new molecular prediction model to predict the outcome of IPF.

Necroptosis is a proinflammatory type of programmed cell death that promotes the release of cellular contents to activate immune response (10–12). More recently, biomarkers of necroptosis including receptor-interacting protein kinase 3 (RIPK3) and mixed lineage kinase domain-like (MLKL) appear to be increased in human IPF samples (13). Necroptosis has been associated with lung diseases such as COPD and ARDS (14, 15). Recently, several studies have started focusing on the relationship between necroptosis and the progression of IPF (13, 16). Necroptosis of alveolar epithelial cells may play a role in bleomycin-induced lung fibrosis in mice (13). However, the association between necroptosis and IPF has not been demonstrated in human IPF samples.

Recently, immune infiltration has been a topic of interest in the field of cancer research. However, immune cells also play a significant role in pulmonary fibrosis (17). Sustained damage to alveolar epithelial cells has been observed to lead to increased apoptosis and induce chronic inflammation with monocyte and lymphocyte infiltration (18). Macrophages play a role in the pathogenesis of pulmonary fibrosis and airway remodeling (19). However, little is known about the landscape of immune infiltration in IPF.

Various immune cells play a significant role in the process of pulmonary fibrosis. When the lung tissue is invaded or injured by pathogens, neutrophils first gather at the damaged site under the action of chemokines such as IL-8 and release pro-inflammatory factors to affect the subsequent inflammatory response (20). In a bleomycin mouse model of pulmonary fibrosis, inhibition of IL-8 function blocks neutrophil aggregation and attenuates the development of pulmonary fibrosis (21). In addition, macrophages and T cells also accumulate in large numbers in fibrotic lung tissue. M1 macrophages are involved in the early inflammatory stage of pulmonary fibrosis by mediating tissue damage and triggering an inflammatory response. In the later stage of pulmonary fibrosis, it is mainly M2 macrophages that promote collagen deposition (22). T cells were abundant in the active fibrosis lesions of IPF patients. In bleomycin-induced T cell deficient mice, extracellular matrix formation was reduced and fibroblast proliferation slowed (23).

Through systematic bioinformatics analysis, the study linked necroptosis with immune infiltration and established a prognostic prediction model based on necroptosis-related genes in IPF. A better understanding of necroptosis and immune infiltration has profound implications for advancing the understanding of IPF.

## Materials and methods

### Data acquisition and process

**Figure 1:**
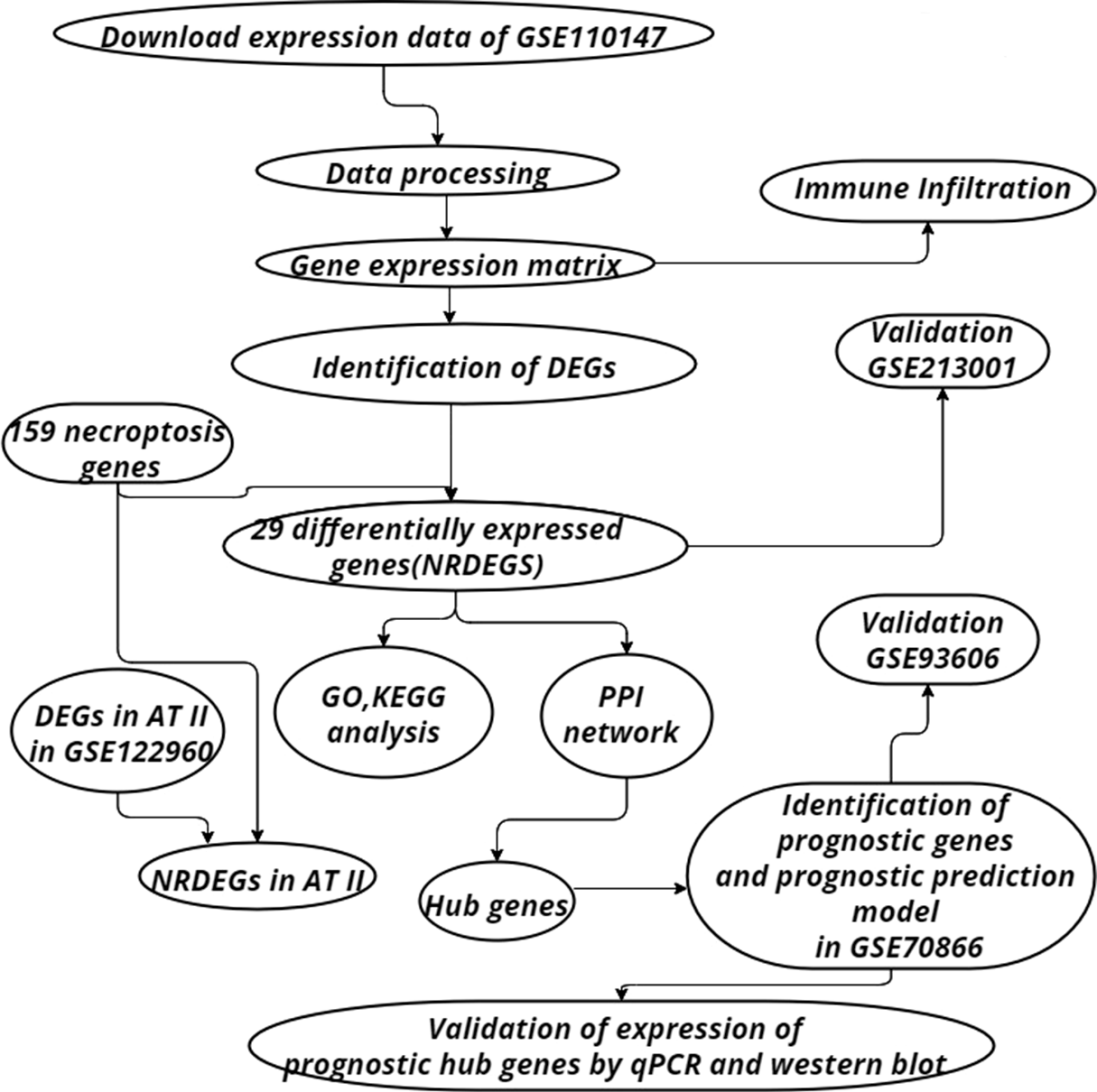
Flowchart of the steps of bioinformatics analysis.

The “GEO query” package of R was utilized to get the expression and the clinical data of IPF and control samples. GSE110147 contained 22 IPF and eleven normal lung tissues, which were performed on the GPL6244 platform. GSE70866 contained sequencing data of BAL cells from 176 IPF patients, which were performed on the GPL14550 and GPL17077 platforms. Single-cell RNA sequencing on lung tissue in GSE122960 obtained from eight transplant donors and nine patients with various forms of pulmonary fibrosis. Validation was achieved utilizing the GSE213001 and GSE93606, which were performed on the GPL21290 platform and GPL11532 platform respectively. GSE213001 included data from 62 IPF samples and 77 healthy controls and was utilized to verify the expression of NRDEGs. GSE93606 included 153 IPF samples and was utilized to examine the effect of the prognostic prediction model. The “sva” package was used to remove batch effects. The workflow chart has been illustrated in **Figure 1**.

### Identification of differentially expressed genes related to necroptosis

Target samples in GSE110147 were classified as IPF and control groups. The “FactoMineR” and “factoextra” packages of R were used for principal component analysis (PCA). “Limma” package 3.52.2 was utilized to perform differential analysis. Differentially expressed genes (DEGs) were obtained by differential analysis of the IPF group and control group samples. The cutoff values of adjust p-value and |fold-change| are 0.05 and 2 respectively (24). Use “ggplot2” and “pheatmap” packages to draw the heatmap and volcanic map of DEGs for visualization. Additionally, it was observed that 159 genes were associated with necroptosis from the KEGG pathway database. A Venn diagram was constructed by utilizing an online tool (http://bioinformatics.psb.ugent.be/webtools/Venn/) to locate the differentially expressed genes of necroptosis (NRDEGs).

### Functional enrichment analysis and identification of hub genes

The NRDEGs were entered into the STRING database to construct the PPI network, and the CytoHubba plugin of Cytoscape software (version 3.9.1) was utilized to locate the hub genes. The Gene Ontology (GO) and Kyoto Encyclopedia of Genes and Genomes (KEGG) pathway enrichment were conducted by package “clusterProfiler”. We performed GO and KEGG enrichment analyses on NRDEGs, only terms with adjust p-value <0.05 were considered statistically significant, and the top 10 enriched terms were visualized using a dot plot.

### Immune infiltration and correlation between NRDEGs and immune cells

The Immune Cell Abundance Identifier (ImmuCellAI) is a tool for estimating the abundance of 24 immune cells consisting of 18 T-cell subtypes and 6 other immune cells from a gene expression dataset (25). The ImmuCellAI tool was utilized to obtain the abundance of immune cell infiltration between IPF and normal controls, depicting the landscape of immune cell infiltration in IPF. Spearman’s test was utilized to study the correlation between immune infiltration and NRDEGs. The “ggcorrpolt” and “ggplotify”R packages were used to determine and visualize correlations between immune cells in the GSE110147. Correlations between NRDEGs and immune cells were visualized by correlation heatmaps. Differences in immune cell abundance between IPF and control groups are shown by grouped violin plots. Associations between immune cells were visualized by a correlation matrix plot.

### Investigation of expression of NRDEGs in type II alveolar epithelial cells in pulmonary fibrosis

Single cell data from 8 lung transplant donors and 9 patients with pulmonary fibrosis were extracted from the single cell RNA sequencing dataset GSE122960, which had been processed by the data uploaders. We obtained DEGs in AT2 of pulmonary fibrosis tissue and control lung tissue, and processed the data using the “dplyr” R package. A Venn diagram was used to visualize the overlap of DEGs in AT2 and 159 necrotic apoptosis-related genes. The “ggplot2” R package was then used to draw the volcanic map for visualization of NRDEGs.

### Identification of prognostic hub genes and construction of prediction model

The expression matrix of hub genes obtained from the previous analysis in GSE70866 was extracted. Subsequently, univariate and multivariate cox regression was performed to screen genes with prognostic differences using the R packages “survival” and “surminer”, and p value<0.05 was considered statistically significant. KM curves and forest plots are used to visualize the results of univariate and multivariate cox regression analyses. Subsequently, the Cox stepwise regression method was used to screen the important genes from hub genes and establish a necroptosis-related prediction model. For each patient, the risk score was the sum of gene expression and corresponding coefficients obtained from a multivariate Cox regression model. Then use the R package “rms” and “pROC” to construct the nomogram and ROC curve to evaluate the predictive value of the prediction model.

### Validation of expression of NRDEGs and effect of prognostic prediction model

The expression matrix of NRDEGs was extracted from GSE213001. Additionally, the expression differences of NRDEGs between IPF and normal samples were calculated by the “ggpurb” package and visualized by the “ggplot2” package. P-value < 0.05 was considered statistically significant. The prediction performance of the prediction model has been tested in GSE93606. The KM curve shows the difference in prognosis between the high-risk group and the low-risk group.

### Preliminary validation of expression of necroptosis-related prognostic genes in IPF A549 cell model

A549 cell line was obtained from American type culture collection (ATCC). We cultured the A549 cells in Dulbecco’s modified Eagle media (DMEM) (Sangon, China) mixed with 10% FBS (Sangon, China). Cells were cultured in a cell incubator with 5% CO2 at 37°C. The study included control and treatment groups. The treatment group was stimulated with recombinant human transforming growth factor β1 (TGF-β1) (MCE, NJ, USA) (20ng/mL), which is a potent fibrogenic agent. After 24 hours of culture, RNA extracted from cells was collected for Quantitative Real-time PCR (qPCR). Relative gene expression was calculated with the equation 2^-∆∆ct.

### Validation of expression of necroptosis-related prognostic genes in bleomycin-induced pulmonary fibrosis mouse model

Male C57BL/6 mice (6-8 weeks of age, average weight 20-25 g) were purchased from Hunan Slyke Jingda Laboratory Animal Company (Changsha, China). After anesthesia, the experimental mice were given bleomycin (5mg/kg) by tracheotomy to induce pulmonary fibrosis, control mice were given normal saline. After 21 days, lung tissue was collected from mice, part of which was used for subsequent western blotting and the other part for HE and Masson staining. Paraffin sections of lung tissue were prepared and stained by HE and Masson according to the kit instructions. The study was reviewed and approved by the institutional review board (Ethics Committee) of the Second Xiangya Hospital, Central South University.

The procedure of western blotting is as follows. Lung tissue is frozen in liquid nitrogen and ground into a powder in a mortar. Powdered lung tissue was lysed in RIPA buffer (Beijing Kangweishiji Biotechnology Company) with protease and phosphatase inhibitors (Selleckchem) and protein quantification was performed using the Bradford method. Subsequently, the protein was isolated on 10% SDS-PAGE gel (Solarbio) and transferred to 0.45 μM PVDF membrane (Merck Millipore, Billerica, MA, USA). After blocking in 5% (M/V) skim milk for 1 hour, incubate with primary antibody overnight at 4°C. The membranes were then incubated with Goat Anti-Rabbit/Mouse IgG (Santa cruz) at 37°C in 1:5000 dilution for an hour. The active bands were identified using an enhanced chemiluminescence kit (Merck Millipore, Billerica, MA, USA). The images were then analyzed using the ImageJ software (National Institutes of Health, Bethesda, MD, USA).

## Results

### Identification of DEGs and NRDEGs between IPF and control

**Figure 2:**
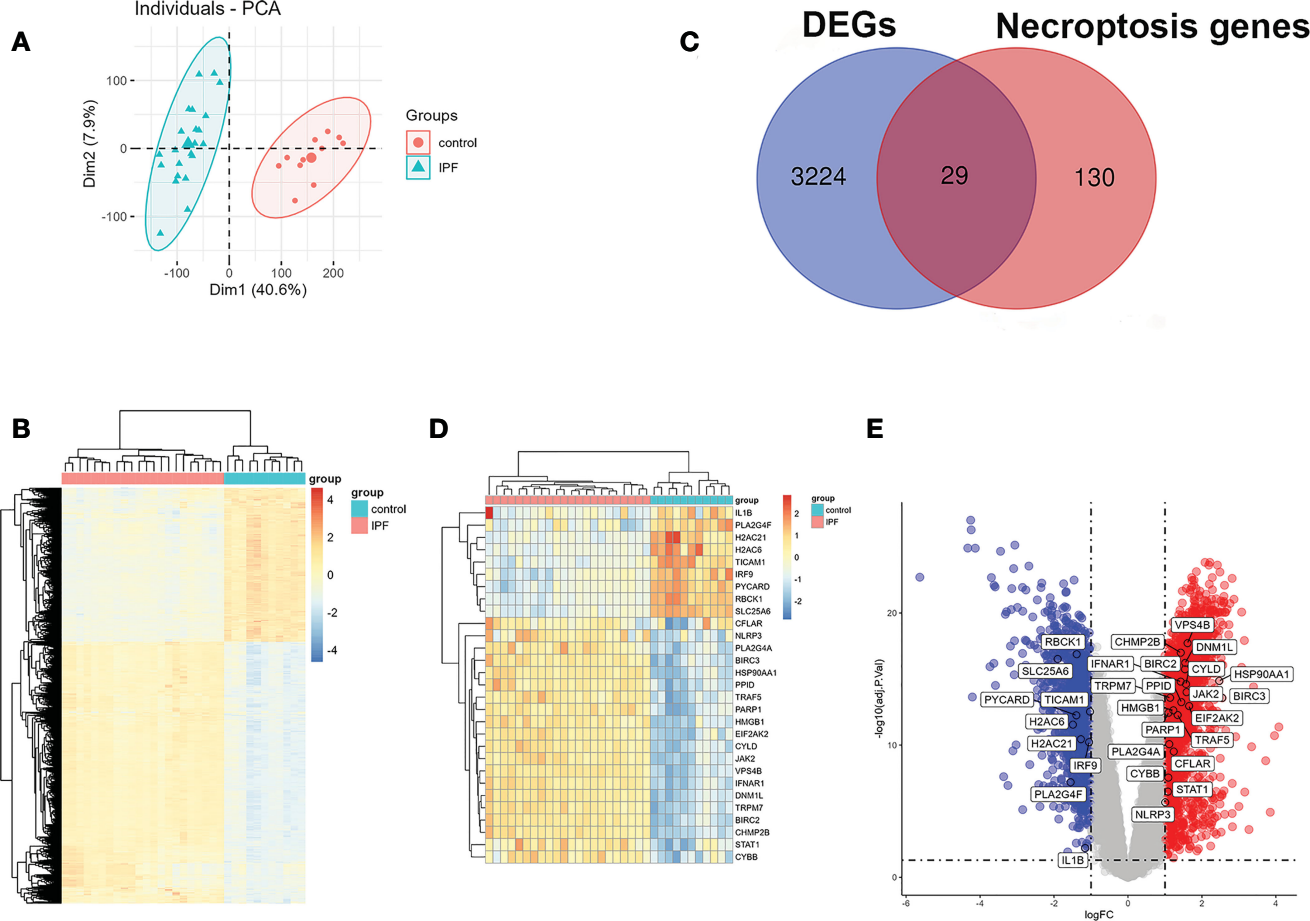
Identification of NRDEGs in IPF. **(A)** The results of the principal component analysis between IPF and control group in GSE110147. **(B)** Heatmap of DEGs from differential analysis of the GSE110147 dataset. **(C)** Venn diagram showing intersection genes between DEGs in GSE110147 and necroptosis-related genes in the KEGG Pathways Database. **(D)** Clustering heatmap of NRDEGs. **(E)** The volcano plot of NRDEGs.

A principal component analysis (PCA) on the IPF group and the control group in GSE110147 was done before performing the difference analysis and it was noted that the comparability between the two groups was obvious. **Figure 2A** depicts the results of PCA. Subsequently, it was observed that 3253 DEGs between IPF and control in GSE110147 were identified by difference analysis. **Figure 2B** shows the expression of DEGs through the visualization of a heat map. The necroptosis-related genes were overlaid with the DEGs in GSE110147, with 29 overlapped NRDEGs being further analyzed. **Figure 2C** shows the 29 NRDEGs in a Venn diagram visualization. The expression of NRDEGs was visualized by heatmaps and volcano plots, as shown in **Figures 2D, E**.

### PPI network and functional pathway analysis

**Figure 3:**
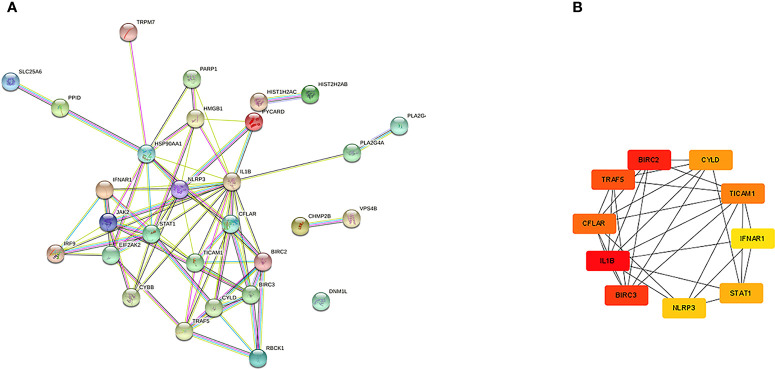
The PPI networks and hub genes were analyzed by the STRING database and Cytoscape software. **(A)** There are 76 edges and 28 nodes in the PPI network. **(B)** Top 10 hub genes explored by CytoHubba.

**Figure 4:**
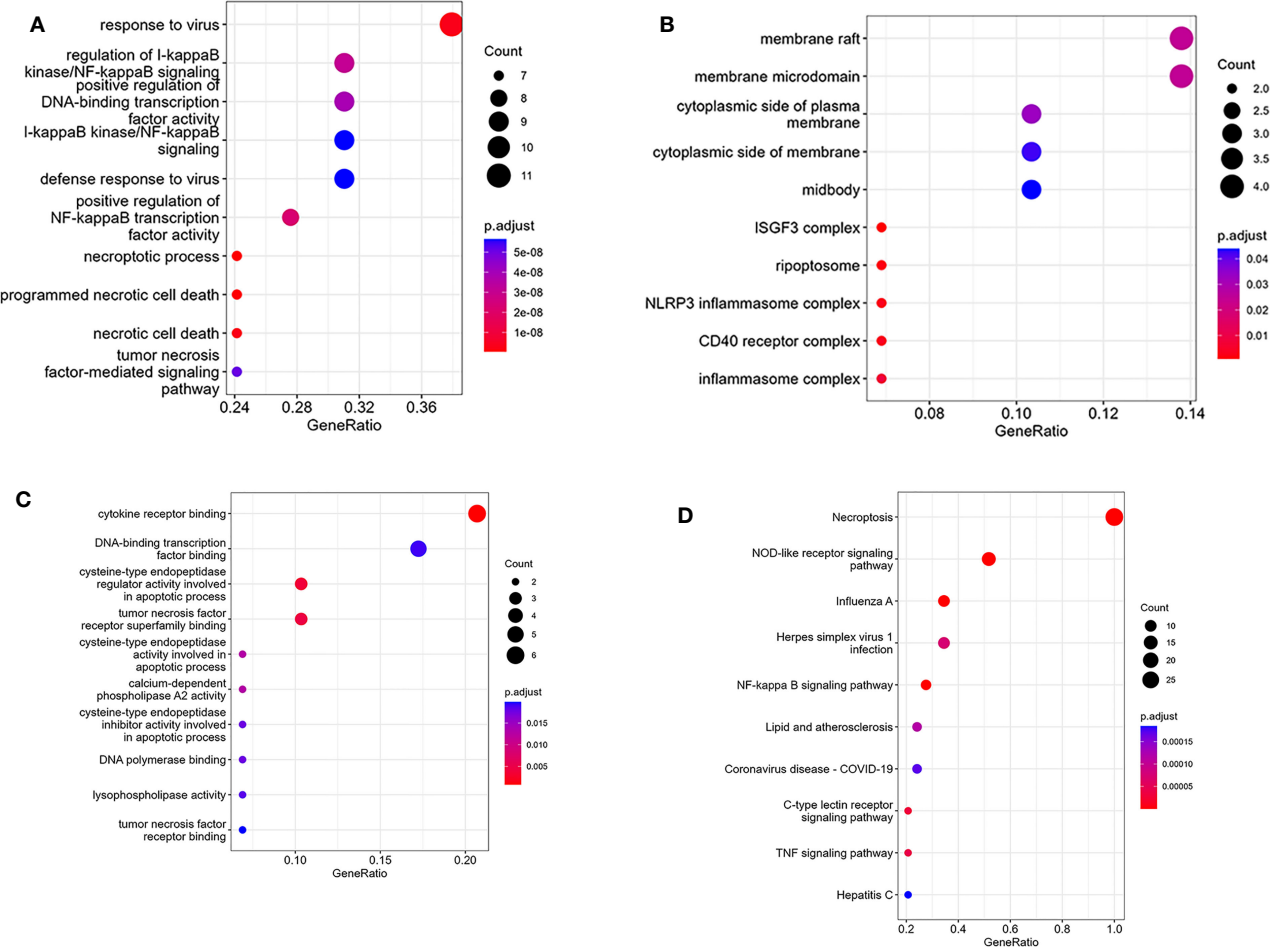
Results of GO and KEGG enrichment analysis using NRDEGs. **(A)** The top 10 GO enrichment analyses of biological processes. **(B)** The top 10 GO enrichment analyses of cellular component analysis. **(C)** The top 10 GO enrichment analyses of molecular function analysis. **(D)** The top 10 GO enrichment analyses of KEGG pathway analysis.

The PPI network of NRDEGs is constructed from the STRING database (**Figure 3A**). An algorithm based on CytoHubba MCC was utilized to identify the top 10 hub genes (**Figure 3B**), including CFLAR, BIRC2, BIRC3, CYLD, TRAF5, IL1B, STAT1, INFAR1, TICAM1, and NLRP3. The GO and KEGG enrichment analyses were done to determine the biological functions and associated signaling pathways of NRDEGs. In total, 1805 associated biological processes and 122 KEGG signaling pathways were identified. The GO enrichment analysis revealed enrichment of defense response to the virus, positive regulation of DNA-binding transcription, regulation of I-kappaB kinase/NF-kappaB signaling, tumor necrosis factor-mediated signaling, necroptotic process, and programmed necrotic cell death in biological processes (**Figure 4A**). Moreover, this study also demonstrated enrichment of the cytoplasmic side of the plasma membrane, membrane microdomain, NLRP3 inflammasome complex, and membrane raft in cellular components (**Figure 4B**). Furthermore, enrichment of tumor necrosis factor receptor superfamily binding, cysteine-type endopeptidase activity involved in the apoptotic process, and cytokine receptor binding was also found in molecular function (**Figure 4C**). The KEGG analysis revealed that NRDEGs were enriched in necroptosis, NOD-like receptor signaling pathway, TNF signaling pathway, NF-kappa B signaling pathway, influenza A, coronavirus disease-COVID-19, herpes simplex virus 1 infection, lipid and atherosclerosis, C-type lectin receptor signaling pathway, and hepatitis C (**Figure 4D**).

### Immune infiltration and correlation between NRDEGs and immune cells

**Figure 5:**
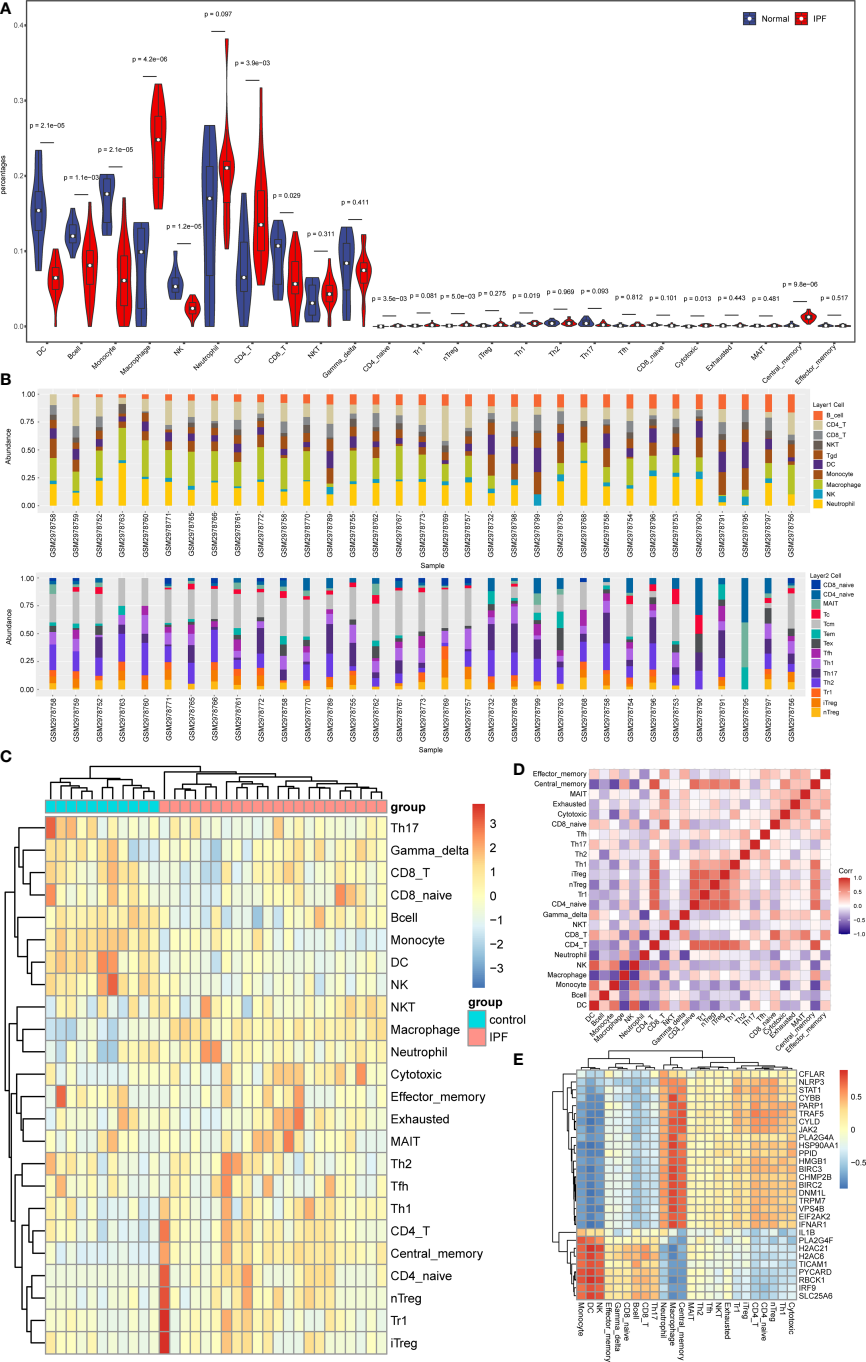
Analysis results of immune infiltration in IPF. **(A)** The Violin plot shows immune cell infiltration and differences between IPF and normal samples. **(B)** Stacked bar chart of the immune cell in all samples. **(C)** Clustered heatmap of proportions of 24 immune cell types. **(D)** The correlation matrix shows the correlation of the proportions of 24 immune cells. **(E)** Heatmap of the correlation of NRDEGs with 24 types of immune cells.

Through enrichment analysis, it was observed that NRDEGs were primarily associated with immune-related signaling pathways. Thus, the association was investigated between necroptosis and immune infiltration in IPF by utilizing the ImmuCellAI algorithm. The result revealed that 12 immune cell types were significantly different between IPF and control groups (**Figures 5A–D**). It was noted that macrophages, neutrophils, CD4 T cells, and nTreg cells were mainly enriched in IPF. However, in the control group, DC cells and NK cells were enriched. Additionally, the results of the spearman correlation test showed that NRDEGs were mainly positively correlated with macrophage and neutrophil infiltration and negatively correlated with DC cell and NK cell infiltration, which were visualized by correlation heat map (**Figure 5E**).

### Investigation of expression of NRDEGs in type II alveolar epithelial cells in pulmonary fibrosis

**Figure 6:**
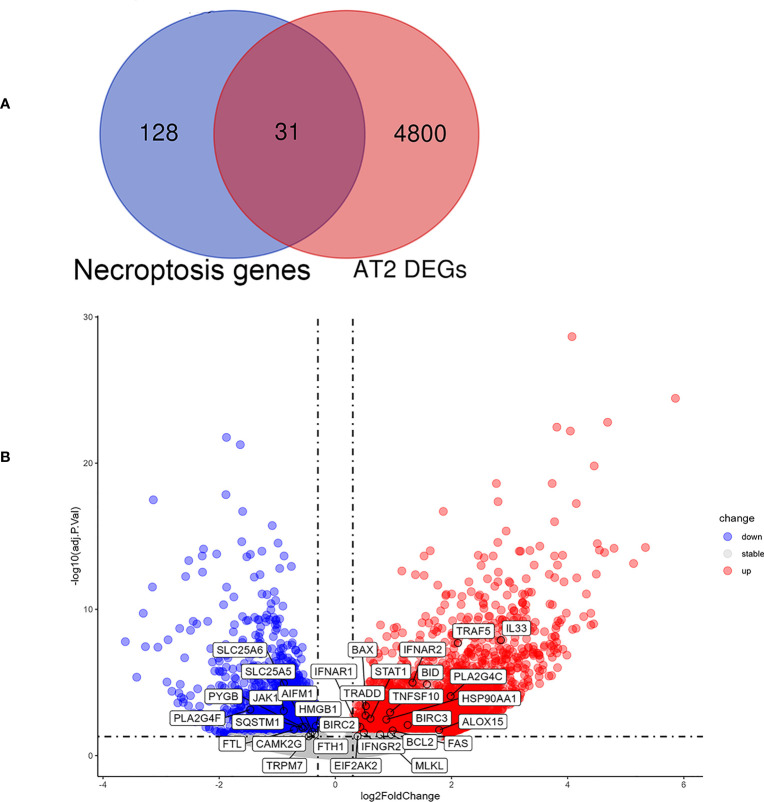
Analysis results of NRDEGs in AT II of IPF. **(A)** Venn diagram showing intersection genes between DEGs in AT II and necroptosis-related genes in the KEGG Pathways Database. **(B)** The volcano plot of NRDEGs in AT II.

In order to further explore the relationship between necroptosis and cell types in IPF, we extracted single cell sequencing data from GSE122960 for analysis. The results showed that the expression of necroptosis-related genes was increased in AT II of fibrotic lung tissue. It was observed that 4831 DEGs between IPF and control in AT II. The 159 necroptosis-related genes were overlaid with the DEGs in AT II, with 31 overlapped NRDEGs being screened, including MLKL, IFNAR1 and TRAF5. **Figure 6A** shows the 31 NRDEGs in a Venn diagram visualization. The expression of NRDEGs was visualized by volcano plots, as shown in **Figure 6B**.

### Identification of prognosis-related hub genes and prognostic prediction model

**Figure 7:**
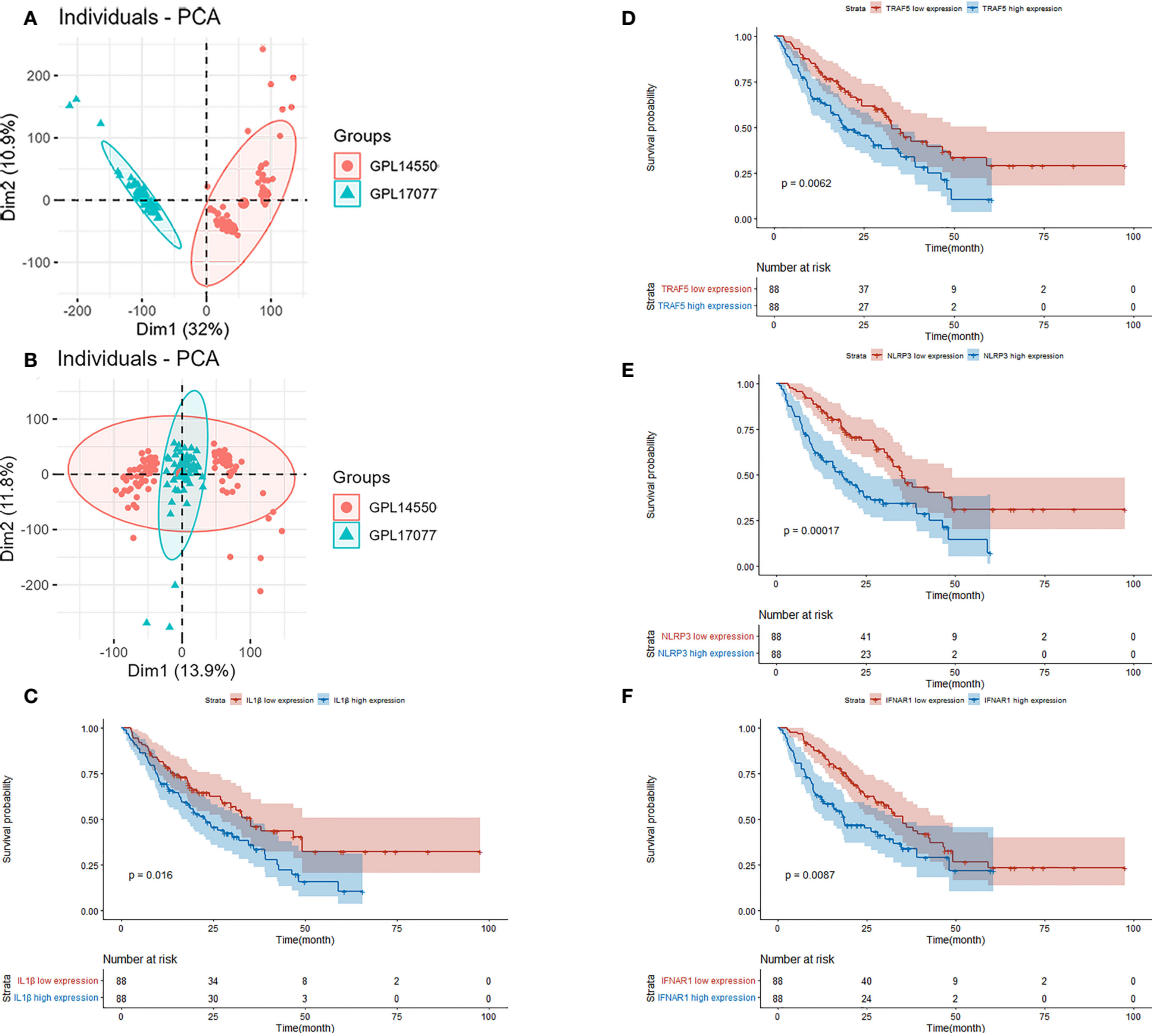
**(A)** PCA plot before removing the batch effect. **(B)** PCA plot after removing the batch effect. **(C)** Kaplan–Meier overall survival plot between IL-1β high expression group and low expression group. **(D)** Kaplan–Meier overall survival plot between TRAF5 high expression group and low expression group. **(E)** Kaplan–Meier overall survival plot between NLRP3 high expression group and low expression group. **(F)** Kaplan–Meier overall survival plot between IFNAR1 high expression group and low expression group.

**Figure 8:**
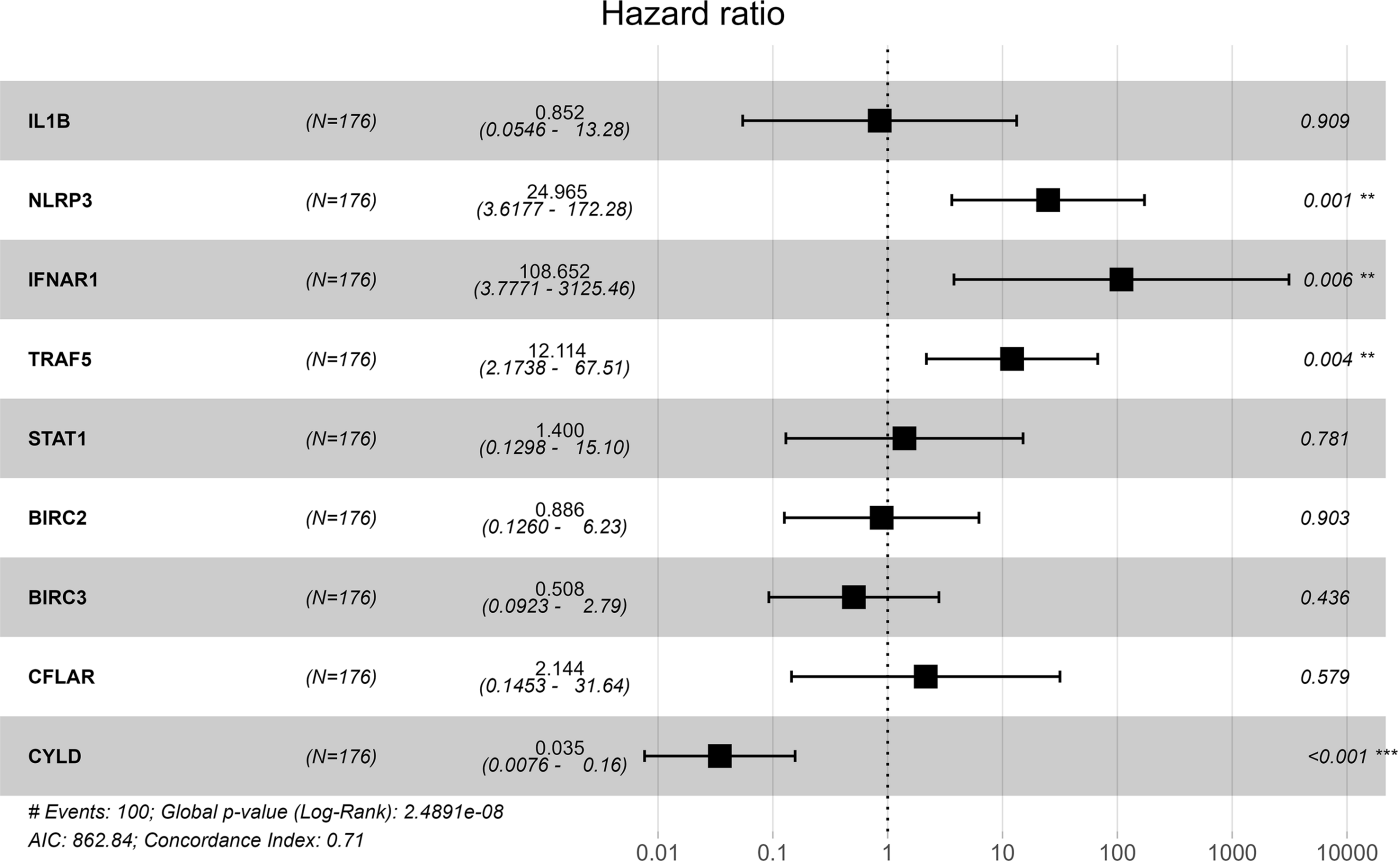
Forest plot of hub genes with P <0.05 by multivariate Cox regression.

**Figure 9:**
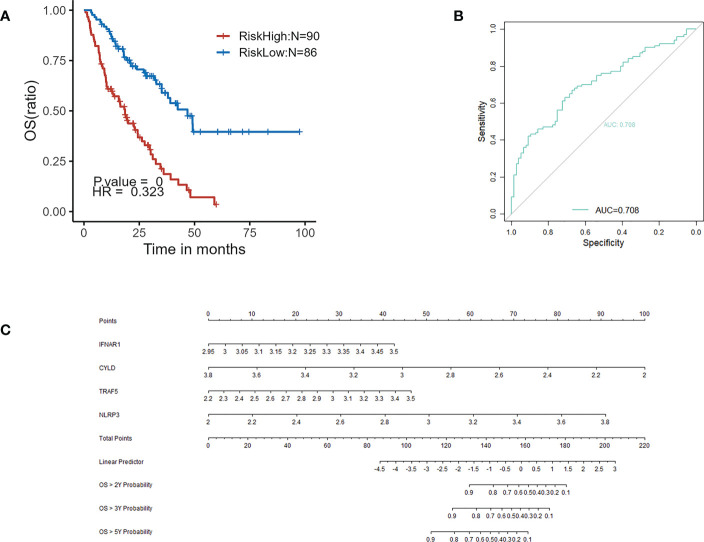
**(A)** Kaplan–Meier plot of overall survival between high-risk and low-risk groups in GSE70866. **(B)** ROC curve evaluated the predictive value of the model for the prognosis of patients in discovery cohort. **(C)** The nomogram for overall survival based on Cox model.

GSE70866 contained sequencing data of BAL cells from 176 IPF patients, and the batch effect was eliminated by the “sva” package. The removal effect is presented as PCA plots (**Figures 7A, B**). The expression matrix of hub genes and prognostic data were extracted and KM curves showed the genes with survival differences obtained by univariate Cox regression analysis (**Figures 7C–F**). Multivariate cox regression showed that IFNAR1, TRAF5, NLRP3, and CYLD were associated with prognosis. The results are displayed in the form of a forest plot (**Figure 8**). The cox stepwise regression method was used to screen the 4 optimal variables from the hub genes related to necroptosis, and then according to the expression levels of the 4 characteristic hub genes and the corresponding coefficients obtained from the multivariate Cox regression model, A risk score was estimated for each patient: risk score = −3.5134 × CYLD expression + 4.9017 × IFNAR1 expression + 3.1992 × NLRP3 expression + 2.2581 × TRAF5 expression. Patients were divided into high-risk and low-risk groups by the median risk score. Compared with the low-risk group, the prognosis of the high-risk group was significantly worse (**Figure 9A**). The ROC curve showed that the evaluation model had good predictive value for the prognosis of IPF patients, with an AUC of 0.708 (**Figure 9B**). This study also established a nomogram for overall survival prediction based on the Cox model (**Figure 9C**).

### Validation of expression of NRDEGs and effect of prognostic prediction model

**Figure 10:**
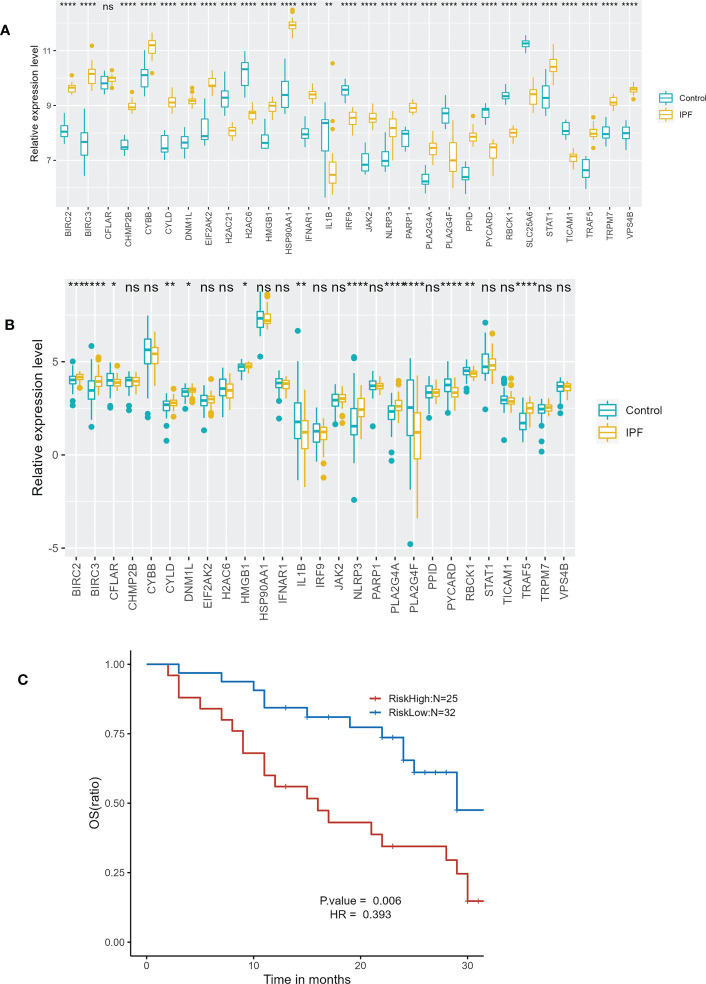
External dataset validation. **(A)** Expression of 29 NRDEGs in GSE110147. **(B)** Expression of 27 identical NRDEGs in GSE213001. **(C)** Kaplan–Meier plot of overall survival between high-risk and low-risk groups in GSE93606 validation cohort. "ns" means p-value >0.05, which is not statistically significant. *p<0.05,**p<0.01,***p<0.001,****<0.0001.

A total of 27 identical NRDEGs were found through the analysis of GSE213001. The expression trends of 12 genes in the 27 NRDEGs were consistent with those in GSE110147, including BIRC2, BIRC3, CYLD, DNM1L, HSP90AA1, IL1B, NLRP3, PLA2G4A, PLA2G4F, PYCARD, RBCK1 and TRAF5 (**Figures 10A, B**). Overall, the validation results in GSE213001 are relatively consistent with GSE110147. We further validate the effect of the prognostic prediction model in the external dataset GSE93606. We divided IPF patients into high-risk and low-risk groups according to the risk score. In the GSE93606, the survival comparison showed that the prognosis of the low-risk group was significantly better than that of the high-risk group (**Figure 10C**).

### Preliminary validation of expression of necroptosis-related prognostic genes in IPF A549 cell model

**Figure 11:**
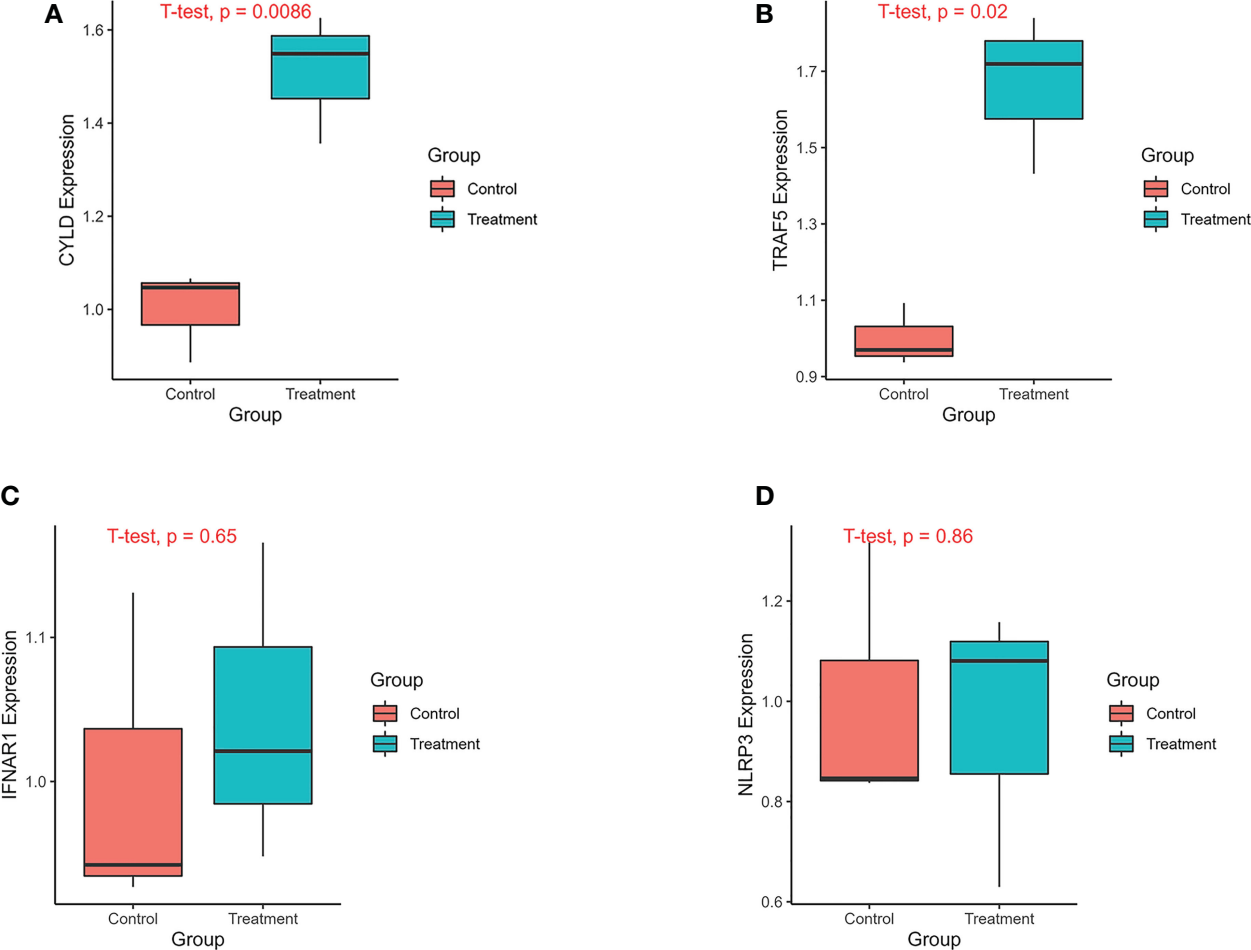
The expression of genes was verified by qPCR. The experimental groups included A549 control group and A549 treatment group. The A549 treatment group was treated with TGF-β1(20ng/ml) for 24 hours. The treatment group and the control group each had 3 replicates. **(A)** The expression of CYLD between treatment and control groups. **(B)** The expression of TRAF5 between treatment and control groups. **(C)** The expression of IFNAR1 between treatment and control groups. **(D)** The expression of NLRP3 between treatment and control groups.

The analysis of previous single cell sequencing data set of pulmonary fibrosis showed that the necroptosis was closely related to AT II. We found that both MLKL, a marker of necroptosis, and necroptosis-related prognostic genes such as IFNAR1 and TRAF5 were highly expressed in ATII. Therefore, we constructed IPF A549 cell model using TGF-β1 to verify the mRNA expression of four key genes in the prognostic prediction model. The results of qPCR showed that CYLD, NLRP3, IFNAR1 and TRAF5 were highly expressed in the TGF-β treatmentgroup, while relatively lowly expressed in the control group. The expression difference of CYLD, and TRAF5 between the control group and the IPF group was statistically significant (**Figure 11**).

### Validation of expression of prognostic genes in bleomycin-induced pulmonary fibrosis mouse model

**Figure 12:**
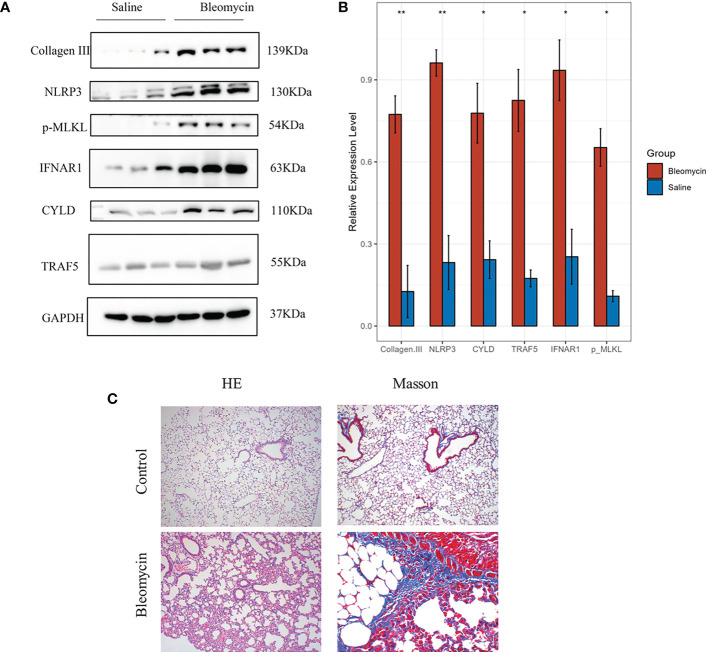
The expression of genes was verified by western blotting in Bleomycin induced pulmonary fibrosis in mice. The experimental group was induced with bleomycin (5mg/kg), while the control group was treated with normal saline. After 21 days, lung tissues were collected for western blotting, HE and Masson staining. **(A)** Protein expression levels of p-MLKL, CYLD, NLRP3, TRAF5 and IFNAR1 in control group and bleomycin group. **(B)** The results of quantitative analysis of gray values of protein expression of these four genes measured by ImageJ (*p<0.05, **p<0.01). **(C)** HE and Masson staining results of control group and bleomycin group.

In order to further verify the results of cell experiments *in vitro* and simulate the pathological process of patients with pulmonary fibrosis as much as possible, we successfully constructed a bleomycin-induced pulmonary fibrosis mouse model for *in vivo* experiments. We were able to study the occurrence of necroptotic events during the pulmonary fibrosis process in bleomycin mouse model of pulmonary fibrosis. Western blotting showed that necroptotic marker p-MLKL and four necroptosis-related prognostic genes, including CYLD, NLRP3, TRAF5 and IFNAR1, were highly expressed in bleomycin induced lung tissues of mice, and the difference was statistically significant (**Figures 12A, B**). The results of *in vitro* cell experiments were further verified by the protein expression level. The results of HE and Masson staining showed that compared with the control group, the bleomycin group had significantly increased lung tissue inflammation and collagen deposition (**Figure 12C**).

## Discussion

Necroptosis plays a significant role in the pathophysiology of lung diseases, like COPD and ARDS. However, its role in IPF is still undetermined. This study, confirmed the presence of necroptosis in IPF, as evidenced by altered expression of necroptosis-associated 29 genes and enrichment of necroptosis-related signal pathways in KEGG. Additionally, KEGG analysis showed that NRDEGs were primarily involved in TNF signaling pathway and NOD-like receptor signaling pathway, which were all immune inflammatory response pathways. Furthermore, significant differences were noted in immune cell content between IPF and normal samples. Interestingly, most NRDEGs had a positive correlation with macrophage, neutrophilic, and CD4 T cell infiltration but a negative correlation with DC and NK cells. More interestingly, we found that the expression of MLKL, a marker of necroptosis, and other genes associated with necroptosis were significantly increased in AT II of IPF, and also significantly increased in fibrotic lung tissue. However, due to the strict setting of our logFC cutoff value, Therefore, RIPK3 and MLKL were not included into the DEGs in the previous analysis of pulmonary fibrosis tissue in GSE110147, so we conducted a follow-up animal experiment for additional verification. These results may suggest that necroptosis in IPF occurs mainly in AT II, and that the release of cell contents in AT II may further cause the aggregation of pro-inflammatory cells. We then constructed IPF A549 cell model and bleomycin-induced pulmonary fibrosis mouse model to further verify and supplement the results of our bioinformatics analysis through *in vitro* and *in vivo* experiments. We detected that the expression of p-MLKL and four other prognostic related genes was significantly increased in fibrotic lung tissue, which laid a foundation for further exploration of the relationship between necroptosis and pulmonary fibrosis. Given the poor prognosis of IPF patients, it is of great significance to establish a prognostic staging system for individualized treatment. In this study, we used transcriptional profiling of bronchoalveolar lavage fluid (BALF) to analyze the relationship between levels of necroptosis-related molecular biomarkers and the prognosis of IPF patients. We established a new prognostic prediction model including 4 gene signatures for IPF patients. This model shows good applicability in an external validation cohort. These findings provide new insights into the relationship between molecular biomarkers involved in IPF progression events and the prognosis of IPF patients. Based on our information, this is the first bioinformatics study to describe the relationship between necroptosis and IPF in human samples.

When the lung gets damaged, ATII activates macrophages and releases many inflammatory factors. Inflammatory factors recruit leukocytes and also induce apoptosis of alveolar epithelial cells. With the further progression of inflammation, a large number of alveolar epithelial cells undergo necroptosis. AT IIproliferates, migrates, and differentiates into AT Ifor tissue repair, the damaged lung tissue then gets repaired (26). When the damage is persistent and greater than the repair capacity of lung tissue, the apoptosis of alveolar epithelial cells and changes in the inflammatory microenvironment occur. The fibroblasts activate, proliferate, migrate, and transdifferentiate into myofibroblasts which are the primary cell type producing the extracellular matrix (ECM) in pulmonary fibrotic lesions and generating contractile collagen types I and III, which are important factors for fibrosis formation. In a mouse model of pulmonary fibrosis, targeted induction of alveolar epithelial cells injury could result in the occurrence of pulmonary fibrosis (27).

Notably, in this study, it was revealed that the necroptosis signaling pathway was enriched in the functional enrichment analysis, which further suggested that necroptosis of AT II may be part of the pathogenesis of IPF. This points out the direction for further exploration of therapeutic strategies for IPF.

We also found some necroptosis-related molecular biomarkers associated with the prognosis of IPF patients, which were involved in the inflammatory immune response. CYLD is a gene encoding a deubiquitinating enzyme that can negatively regulate the NF-κB signaling pathway. Furthermore, CYLD is involved in regulating an alternative pathway to tumor necrosis factor-mediated necroptosis that is not controlled by NF-κB signaling. Instead, this pathway relies on the kinase activity of RIPK1 and is mediated by tumor necrosis factor receptor complex IIb. CYLD regulates the formation of the complex IIb (28, 29). TRAF5 is an important signal transduction molecule of a member of the tumor necrosis factor receptor superfamily, and the main pathways mediated are the classical and alternate NF-κB activation pathways, as well as the MAPK and JNK activation pathways. The NLRP3 inflammasome is a cytoplasmic multiprotein complex composed of NLRP3, ASC, and pro-caspase-1. The NLRP3 inflammasome is tightly linked to cell death, including pyroptosis, necroptosis, and ferroptosis (30). NLRP3 inflammasome can activate the IL1/NF-κB pathway to induce an inflammatory response, which eventually leads to necroptosis in IPF. GO and KEGG enrichment analysis identified several other biological pathways that are relevant to IPF. TNF signaling pathways might promote extrinsic apoptosis and necroptosis. The necroptosis associated with TNF signaling pathways and NOD-like receptor signaling pathways has been extensively studied (31). The overexpression of these genes and activation of related pathways may be related to the poor prognosis of IPF. This study analyzed and identified these characteristic genes and related signaling pathways, providing new ideas and insights into the prognosis of IPF.

In this study, ImmuCellAI was applied to further explore the landscape of immune infiltration in IPF. IPF had higher scores for macrophages and neutrophils, the most abundant immune cells in the lung, a finding consistent with previous findings (19, 32). Macrophages played a central role in tissue repair, immunity, and airway remodeling in pulmonary fibrosis. Acute lung injury promotes the expression of M1-type macrophages, and proinflammatory cytokines are highly expressed in the early inflammatory stage of pulmonary fibrosis (33–35). However, a persistent inflammatory response would act as a trigger for the fibrotic response in the lungs. M2 macrophages could produce profibrotic mediators to promote myofibroblast proliferation and activate fibroblasts (36–38).

Furthermore, T cells are the most important of all immune cells in mediating immune responses. They are widespread in areas of active disease in the IPF lungs (39–41). Historically, an imbalance of Th1/Th2 immune responses has been considered central to the pathogenesis of IPF (42–44). Our results also confirmed the existence of a Th1/Th2 imbalance in IPF. The study revealed that necroptosis is closely associated with the infiltration of immune cells. Thus, necroptosis might promote the progression of IPF by activating immune infiltration.

There are some limitations to this study. First, large-scale clinical trials are needed for mechanism research. Second, the sample size is relatively insufficient. Third, this study was unable to determine if necroptosis induced the infiltration of immune cells, nor it could confirm if immune cells were also involved in the process of necroptosis of AT II. Further studies should be conducted to elucidate the underlying mechanism.

## Conclusions

The expression levels of necroptosis-associated genes in IPF and control differed significantly, according to bioinformatics analysis. Furthermore, a correlation between the infiltration of multiple immune cell types and necroptosis in the lung tissue of patients with IPF, and differences in immune cell infiltration between normal lung tissue and IPF lung tissue were found. The association between AT II and necroptosis in fibrotic lung tissue was found through single-cell sequencing data analysis, because the expression of markers of necroptosis and necroptosis-related genes was significantly increased in AT II of fibrotic lung tissue. In addition, we explored the prognostic molecular biomarkers related to necroptosis, and the prognostic prediction model based on multiple signature genes provides a new way to predict the progression and prognosis of IPF. Thus, this study has paved the way for further exploration of the necroptosisinIPF. Additionally, efforts should be taken to develop new therapeutic targets.

## Data availability statement

The datasets presented in this study can be found in online repositories. The names of the repository/repositories and accession number(s) can be found in the article/supplementary material.

## Ethics statement

The animal study was reviewed and approved by The institutional review board (Ethics Committee) of the Second Xiangya Hospital, Central South University.

## Author contributions

ZX designed this study, BQ, WW, and LG were responsible for data acquisition and filter, YC and JW prepared the interpretation, HC performed data analysis and preparation for the drafting of the manuscript, YY revised the manuscript and oversaw the study. All authors contributed to the article and approved the submitted version.
